# Couplants in Acoustic Biosensing Systems

**DOI:** 10.3390/chemosensors10050181

**Published:** 2022-05-09

**Authors:** Rayyan Manwar, Loїc Saint-Martin, Kamran Avanaki

**Affiliations:** 1 Richard and Loan Hill Department of Bioengineering, University of Illinois at Chicago, Chicago, IL 60607, USA; rmanwar@uic.edu (R.M.); lsaint2@uic.edu (L.S.-M.); 2 Department of Dermatology, University of Illinois at Chicago, Chicago, IL 60607, USA

**Keywords:** acoustic sensors, coupling agent, couplant, liquid/gel, semi-dry, dry

## Abstract

Acoustic biosensors are widely used in physical, chemical, and biosensing applications. One of the major concerns in acoustic biosensing is the delicacy of the medium through which acoustic waves propagate and reach acoustic sensors. Even a small airgap diminishes acoustic signal strengths due to high acoustic impedance mismatch. Therefore, the presence of a coupling medium to create a pathway for an efficient propagation of acoustic waves is essential. Here, we have reviewed the chemical, physical, and acoustic characteristics of various coupling material (liquid, gel-based, semi-dry, and dry) and present a guide to determine a suitable application-specific coupling medium.

## 1. Introduction

Biosensors are analytical devices consisting of sensing elements that are used to detect the presence or concentration of a biological analyte, such as a molecule, a subcellular structure, or a microorganism [1,2]. Depending upon various types of signals transduced, biosensors can be categorized as follows: electrochemical, electromagnetic (optical, thermal), and acoustic [3,4]. Since the abundance of biomarkers in human fluid is low compared to that of irrelevant biomolecules, a high-differentiation ability is required for electrochemical biosensors. Moreover, the matrix effect caused by biomolecules, other than the target, interferes with the target recognition process, which leads to an increased probability of false-positive results [5]. This phenomenon necessitates the electrochemical biosensing mechanism to be mostly invasive in nature. Optical and thermal biosensing techniques can be non-invasive with high sensitivity; however, they have a low penetration depth due to the rapid dissipation of optical/thermal energy through highly scattered tissue media [6].

On the other hand, acoustic biosensing is a popular technique due to advancements in ultrasound detection technologies that are non-invasive and enable deeper penetration [7] with reasonable resolution and sensitivity.

**Figure 1:**
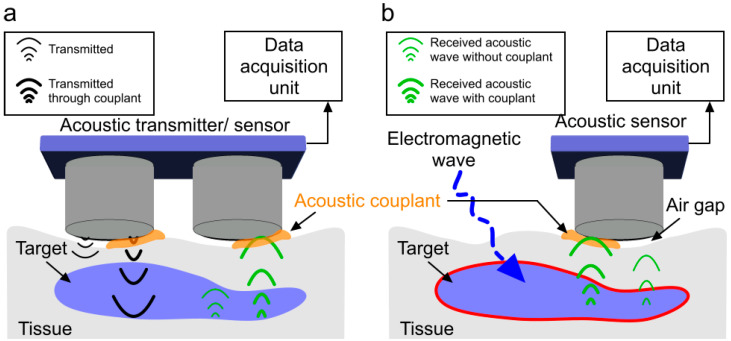
All-acoustic sensor versus induced-acoustic sensor with and without a couplant. (**a**) Acoustic wave transmission and reception in an all-acoustic sensor; (**b**) acoustic wave detection in an induced-acoustic sensor. Inefficient acoustic wave propagation in air is due to a high acoustic impedance mismatch between the tissue and the transducer. Acoustic coupling medium reduces the impedance mismatch between probe surface and target for improved acoustic wave transmission and reception with decreased loss of signal (thick acoustic waves-green).

Acoustic sensing is based on the transmission and reception of acoustic pressure waves. Acoustic sensors are used to receive acoustic waves induced by the transmitted ultrasound (e.g., ultrasound transducers) or electromagnetic waves (e.g., in electroacoustic [8,9], magnetoacoustic [10,11], thermoacoustic [12,13], photoacoustic [7,14,15,16,17,18,19,20,21,22,23,24,25,26,27,28,29,30,31,32,33], X-ray acoustic [34,35], and proton-acoustic [36,37] modalities) (see Figure 1). Acoustic biosensors employ, for instance, the piezoelectric effect to excite acoustic waves electrically to an input transducer and to receive waves at the output transducer. The incident acoustic pressure waves deform piezo material, and they are measured in terms of the potential difference across the piezo electrodes induced by the deformation [38,39]. Table 1 summarizes the advantages and disadvantages of different types of biosensors.

Acoustic sensing is performed efficiently in the frequency range from several kHz up to several gigahertz. Acoustic waves (ultrasound) are directional, making them more sensitive to the density and elastic properties of the materials they pass through [44]. Acoustic sensors are popular due to their low cost, simplicity, availability, and clinical acceptance [45,46,47]. In addition to biomedical applications, they have been widely used for non-destructive testing [44,48,49].

**Figure 2:**
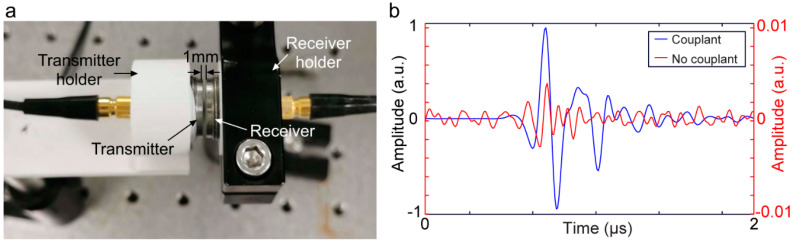
Impact of couplant on acoustic signal propagation. (**a**) Experimental setup demonstrating a pair of ultrasound transducer (transmitter and receiver) coaxially placed. The distance between the transmitter and receiver was 1 mm; (**b**) normalized acoustic signal strength acquired by the receiver in (**a**) the presence of coupling medium (ultrasound gel-blue line) and no couplant (air gap-red line).

Every material presents an impedance to the passage of acoustic waves. The specific impedance of a material is determined by its density and speed of sound (governed by Young’s modulus and elasticity) through the material. The speed of sound can be calculated from the elastic modulus and density [50]. For the maximal transmission of energy from one medium to another, the impedance of the two media should be equal. In a bioacoustic sensor, with air coupling, the acoustic wave is poorly transmitted through the air and mostly reflected before reaching the target due to a high acoustic impedance mismatch. Considering that the unit of acoustic impedance is the MPascal second per cubic meter, represented in MRayl, the acoustic impedance of air is ~10^−4^ MRayl and that of a biological tissue is ~1.63 MRayl [51]. This indicates that even a small air gap between the acoustic sensor and the target will cause a reduction in acoustic waves by 99.998%. Therefore, the presence of a coupling medium is essential for efficient propagation by displacing air and filling contours, which brings the target and transducer impedance closer to equality. The coupling medium is called an acoustic couplant. Figure 1 shows the effect of not using acoustic couplants in an acoustic sensing experiment. In this demonstration, the transducer surface is partially (only right half) covered with acoustic couplants. Therefore, stronger transmitted and received acoustic pressure waves is observed through the couplant. The left half of the transmitter and receiver is not covered with acoustic couplant (causing an airgap) and corresponding acoustic pressure wave is shown to be weak (due to the high attenuation originating from a high acoustic impedance mismatch). A simple experiment was conducted using two acoustic transducers (C539-SM, Olympus, Center Valley, PA, USA, aperture: 1.25 cm, frequency: 1 MHz) as a pair of transmitter and receiver to demonstrate the effect of the acoustic couplant. The experimental setup is shown in Figure 2a. The transducers were coaxially placed (gap: 1 mm). A 10 V peak–peak sinusoidal signal was applied to the transmitter that was attached to the phantom without any couplant. The received signal was acquired by Picoscope-5242D (sampling frequency: 100 MHz, 15 bit, Pico Technology, Tyler, TX, USA) in two different scenarios: (1) gap between the receiver and the phantom was filled with ultrasound gel (couplant) and (2) in air (no couplant). The impact of the couplant to enable efficient propagation is as follows: the acquired signal strength of the experiment with couplant is ~250 times higher compared to the signal strength when the experiment was performed in air (see Figure 2b).

An ideal couplant is the one with the least absorption of acoustic energy or a small distortion of its path. Ideally, the coupling medium should be fluid so as to fill all available spaces, be relatively viscous so that it stays in place, have an impedance appropriate to the media it connects to, have a low reflection coefficient, have least air trapped, nontoxic, and should allow the transmission of acoustic waves with minimal attenuation over a wide operating frequency range [52]. A variety of substances such as water, gel, and oil can be used as acoustic couplants; however, for the best results, it is necessary to use specially formulated couplants. In medical applications, couplants are required to be conformable, have a certain degree of elasticity and adhesion, and must be biocompatible with the target area. In addition, the couplant may also act as a cooling agent to reduce the heat generated from the continuous operation of the sensors. Here, we study the acoustic characteristics of liquid/gel, dry, and semi-dry couplants and describe their main advantages and disadvantages. Table 2 demonstrates an example of each of the types of couplants.

Acoustical properties are those that govern how a medium responds to acoustic waves. While propagating through a medium, acoustic waves experience attenuation due to absorption and scattering and reflection due to acoustic impedance mismatch at the interfaces of a multi layered medium. These phenomena can be realized from acoustic wave propagation theory in visco-elastic medium. Acoustic wave propagation originates from the excitation, induced by transmitted ultrasound (US) or electromagnetic waves, that is incident upon the surface particles of the medium and propagated by particle vibration through the medium. This wave propagation may be described by Newtonian mechanics and stress–strain relations. A detailed explanation of the wave propagation model can be found in [53,54,55]. Here, we briefly define acoustic pressure absorption, scattering, and impedance, which form the basis of acoustic wave characteristics in terms of attenuation. The attenuation factor (due to absorption), which leads to exponential damping of the wave amplitude as a function of depth and frequency, is given by the following:

(1) $$\alpha =\frac{\omega^{2}\eta }{2v_{p}}$$

**Figure 3:**
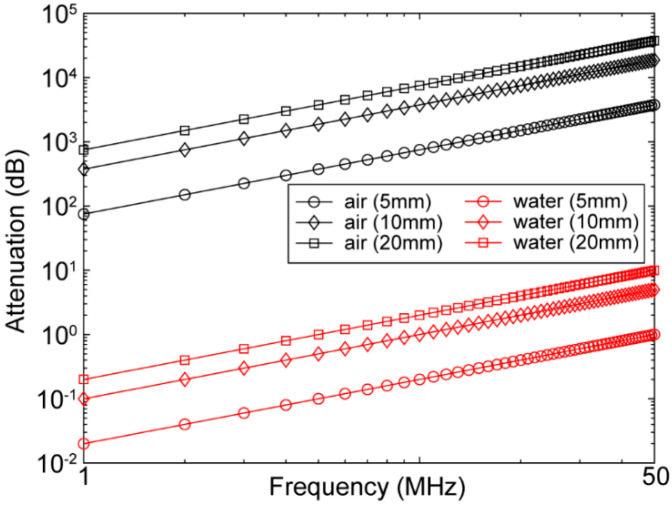
Attenuation of acoustic wave as a function of frequency (1–50 MHz) at different distances (5, 10, and 20 mm) in air (black) and water (red).

Here, ω represents the angular frequency, $v_{p}$ is the phase velocity, η is viscosity, and ρ is the density of the medium. The theoretical attenuations of acoustic waves due to absorption at different frequencies and thicknesses for water and air, based on the attenuation power law [56], are plotted in Figure 3. Attenuation values of air and water are presented in Table 3 [57,58,59]. Moreover, attenuation due to scattering also depends on the thickness of the medium, as well as medium structural discontinuity, grain boundary, inclusions, and voids (air bubbles). On the other hand, acoustic reflection due to acoustic impedance mismatch depends on the medium’s density, Young’s modulus, and elasticity. The acoustic impedance (the resistance exerted by tissue to the sound propagation) can be represented as follows:

(2) $$Z=\rho \omega /k≅\rho v_{p}$$

where k=β+iα represents the wave number and β is the propagation constant. When the impedances of the adjacent materials are known (*Z*_1_ and *Z*_2_), the fraction of the incident wave intensity that is reflected can be calculated with the equation below.

(3) $$R={\left(\frac{Z_{2}-Z_{1}}{Z_{2}+Z_{1}}\right)}^{2}$$

The result from Equation (3) is known as the reflection coefficient, *R*. The amount of energy reflected as a percentage of the original energy can be extracted by multiplying the reflection coefficient by 100.

## 2. Material Collection Method

The data for this review study were collected from published articles, industrial databases, and granted patents. Initially, we searched for available couplants. We used “acoustic couplant” and “coupling agent” keywords for our search. The result was 82 publications in Google Scholar database (October 2021). We then narrowed down our search to only sensing/diagnostics applications of acoustic sensors by adding the keyword “ultrasound” to and excluding the keyword “treatment coupling agents” from the search. We found 4 review articles on this topic; however, they only explored liquid and gel-based coupling agents (i.e., water, hydrogel, mineral oil, and petrolatum). These review articles were published between 2000 and 2004. Since then, more research works have been performed to improve the quality of the liquid and gel-based coupling agents. Alternatively, dry and semi-dry couplants have been introduced to overcome the existing limitations of liquid and gel-based couplants. Here, we have discussed three major coupling agents, (i) liquid/gel-base, (ii) dry, and (iii) semi-dry couplants, their composition, acoustic properties, advantages, and disadvantages in biosensing applications.

## 3. Acoustic Coupling Agents

### 3.1. Liquid/Gel-Based Couplants 

In biomedical applications of acoustic sensors, the main objective of the couplant is to reduce acoustic impedance mismatches between two different materials. Acoustic impedance is dependant on density and longitudinal speed of sound through the propagating medium. Moreover, the absorption and scattering within the medium determines the acoustic amplitude attenuation as a function of frequency and depth. Hence, a low attenuating medium is preferred. In Table 3, we list a number of materials with their respective properties, including some of their acoustic properties.

Many fluids and water-based gels have been used as ultrasound couplants over the years [60,61,62]. The most-used acoustic coupling agents are water, gel, mineral oil, and white petrolatum [52]. A list of liquid/gel-based couplants with their acoustic properties is given in Table 4 [63,64]. In spite of having similar impedance and attenuation characteristics, only gel-based couplants have the additional quality of higher viscosity, which increases couplant adherence to surfaces. Gel viscosity is primarily derived using two different methods: a mixture of copolymer of methyl vinyl ether, maleic anhydride, and a carboxy polymethylene polymer or carboxy polymethylene polymers with hydroxyalkyl cellulose and a polyalkylene glycol [65].

Despite the advantage of liquid/gel-based couplants possessing low acoustic impedance mismatch with the biological tissue, they are amorphous fluids that tend to fall off or dry out over time [66,67]. In addition, liquid and gel couplants may sometimes lead to corrosion when used for a long time and modify the mechanical properties of the transducer [68]. Another issue is that gels could lead to potential bacterial growth if not cleaned properly [69]. In addition, gel and liquid couplants dry, leak, and/or ooze out from the transducer’s sensing surface and cause signal degradation. In clinical applications, the thickness of the gel applied to the imaging area is non-uniform and can easily trap air bubbles (umbrella artifact), becomes messy, and is not comfortable. Having to reapply acoustic couplant during an experiment is problematic because the procedure disturbs the positioning of the probe and takes time. Therefore, liquid/gel-based couplants provide only a temporary or short duration of suitable acoustic transmission/reception and simultaneously contribute to the inconsistencies in the received signal [70]. Bubbles trapped inside water or gel can be removed by keeping the couplant in a vacuum chamber under high pressure. After 20–30 s in a vacuum chamber, air bubbles migrate to the top surface and due to the negative pressure and start to breakdown.

Of the companies that produce acoustic couplants, the majority produce liquid/gel-based couplants (Aquasonic, EMS, KY gel, JPM, PhysioMed, SKF, Biofreeze, University of Hertfordshire, Hertfordshire, UK). There is not a significant clinical difference among these products [71,72]. However, these ultrasound couplants are produced in different atmospheric/mechanical state or form factor for various operating conditions. For specialized applications, companies such as Echo Ultrasonics [73], Magnaflux [74], and Olympus Co. [75] manufacture couplants designed for extreme temperature ranges (−23–538 °C) or acoustic characteristics (e.g., shear wave). These couplants are available as powders or pre-mixed.

### 3.2. Dry Couplants

Dry couplant is mainly used during the development and non-destructive testing [76] of biomedical instrumentation. For dry couplant, an appropriate material selection is crucial such that the couplant can easily be applied to and removed from the imaging area surface, flexible yet self-supporting, and conforms readily to the contours of the surface. In addition, high uniformity in thickness and having the least acoustic impedance mismatch are two other factors for dry couplants.

Dry couplants are made of polymers [77] and developed based on the desired acoustic, mechanical and thermal behavior of the couplant. Polymers are categorized as (1) thermoplastic, (2) thermoset, and (3) elastomers. Thermoplastic polymers are composed of long chains produced by joining small molecules or monomers; they behave in a plastic, ductile manner and soften at elevated temperatures [77]. Some of the most common thermoplastics are low- and high-density polyethylene (respectively, LDPE and HDPE), polypropylene (PP), polyvinyl chloride (PVC), and polystyrene [78]. Thermosetting polymers are composed of long chains of molecules that are strongly cross-linked to each other to form a three-dimensional network structure. These polymers are stronger yet more brittle than thermoplastics. Thermosets can further be classified into rubber-based and hard materials. The most common types of thermosets are epoxide and polyester resins (i.e., polyurethanes, and polyamides) [79].

The most popular dry couplants are the elastomers. In elastomers, some of the cross-linking of the chains are allowed to occur for tuning elastic properties [77,80]. Elastomers have similar acoustic properties to liquid/gel-based couplants and are produced in two forms, flexible or rigid, depending on the requirements of the application they are used in [81]. Elastomers are preferred in imaging applications, due to their stability and structural support, while still having similar acoustic characteristics to liquid or gel-based couplants, specifically low attenuation and impedance matching [66,82]. The acoustic properties of conventional polymers used as dry couplants are summarized in Table 5.

Although a high clamping pressure is applied to the dry coupling material to cause it to slightly deform at the interface in order to couple with the target surface and expel any air trapped between them [87], due to the rigidity of the thick coupling material completely removing air trapped when working with dry couplants is difficult.

Innovation Polymers Inc. (Kitchener, ON, Canada) [88] has developed several dry acoustic couplants among which Aqualene and ACE belong to thermoset and thermoplastic polymer categories, respectively. Aqualene and ACE are based on divinyl olefins with variations in the curing processes and additives to adjust hardness, attenuation, and acoustic velocities. Variation in characteristics can also be originated from differences in their respective forming processes. The forming process can be by injection molding or compression or transfer molding [89]. Different injection pressures and injection-head temperatures are also factor into the change in acoustic and mechanical properties. The properties of these materials used in medical phantom applications as a tissue-mimicking material have been discussed in [90] and further acoustic property analysis has been carried out in [89]. The acoustic properties of commercially available dry couplants are summarized in Table 6.

### 3.3. Semi-Dry Acoustic Couplants

Semi-dry couplants are considered as enclosed assemblies consisting of a thin inflatable membrane made of a dry couplant with a liquid/gel-based couplant (see Section 3.1) inside [91]. Semi-dry couplants are more flexible for avoiding air trapping compared to dry couplants, yet they have a reduced coupling impedance mismatch and attenuation similar to liquid/gel-based couplants. Semi-dry couplants are categorized based on their form factor including patch style, sheet form, pouch, and other customized enclosed assemblies.

Inflatable membranes are typically made of elastomers among which hydrogel (hydrophilic elastomer) is the most popular. Cross-linked hydrophilic elastomers are described as macromolecular networks that swell but do not dissolve in water [60,92,93]. By placing them in water, they hydrate, where they are capable of absorbing large quantities of water (in some cases up to 95% of their own wet weight). Hydrogels may consist mainly of water, yet they are in solid form and dry. The ability of a hydrogel to absorb water arises from hydrophilic functional groups attached to the polymeric backbone. Over time, the polymers will absorb water until they reach equilibrium [94,95]. The composition of the hydrogel depends on the polymer percentage, alkali salt contents, and type of chemical modifiers (viscosifying agent and hydrating agent). Conventional ingredients for developing a hydrogel is as follows: a mixture of 3 to 4% polyvinyl alcohol, 30 to 35% polyvinylpyrrolidone, and hydric alcohol, such as propylene glycol or glycerol in the amount of 20 to 25%. The remaining component is water. When these reactants are heated to 125 to 130 °C followed by cooling and subsequent casting on a suitable release mold, an adhesive hydrogel forms [96]. 

Larson et al. [97] developed a disposable, flexible, elastic sheath type membrane to enclose gel. The flexible sheath comprised a hydrophilic block copolymer and 20 wt.% to about 95 wt.% biocompatible liquid. The hydrogel sheaths are produced with a preferred controlled thickness of 0.05 to 4.0 mm throughout. When in contact with the skin, the sheath becomes lubricous and that provides more adhesion and, therefore, less chance of any air trapping. Sieverding et al. [98] proposed an electroconductive, water-insoluble, hydrophilic, elastomeric pressure-sensitive adhesive patch. This adhesive comprised gel of polyvinylpyrrolidone cross-linked by ionizing radiation, polyalkylene glycol plasticizer, water, and salt selected from ammonium acetate, magnesium acetate, or magnesium sulfate. Richardson et al. [99] claimed the use of hydrophilic material (75–90% water) for the coupling of an ultrasonic probe. Bourne et al. [100] also utilized hydrophilic polymers as acoustic couplants; however, they were intended for non-destructive testing applications. Other than hydrogels, researchers have also developed semi-dry couplants based on polymers (such as polypropylene, polyurethane, and polyethylene). Shikinami et al. [101] proposed a flexible plate-like acoustic coupler. The main component of this couplant is a polyurethane gel of 10 mm thickness and 1.1–2.0 MRayl acoustic impedance. The film layer that covers the gel and is exposed to skin is made of polypropylene of 0.1 mm thickness and 0.2–5.0 GPa Young’s modulus. The overall acoustic impedance of the coupler was 1.6–5.0 MRayl. Murdock et al. developed a fluid (i.e., water/gel) medium within a flexible membrane made of Polyethylene Terephtalate (PET), also known as Mylar. To reduce reflection from the membrane and the coupling fluid, the thickness of the PET layer was kept to only 25.4 µm. Jahnke et al. developed an enclosed rigid container for holding ultrasound gel. The rigid container was fabricated with highly compliant elastic materials, such as silicone, polyurethane, latex, and rubber, on superior and inferior surfaces. The container was filled with liquid couplant such as degassed water or gel. The container includes vents with caps for filling or emptying the acoustic coupling medium. Lima et al. proposed a low-cost solution to semi-dry couplants by using latex and nitrile glove material to hold water inside. Unlike conventional semi-dry couplant configurations, Pretlow et al. developed a gel pad without any membrane made from cellulose that provides rigidity with a mixture of glycerin and water/oil that provides flexibility and adhesion. The gel pad was relatively thin (approximately 1.5 mm) and could easily be positioned between an ultrasound transducer probe and the specified area of a patient. Buchalter et al. developed a similar yet disposable ultrasound coupling pad that adheres to a patient’s skin. Silicone elastomer and sylgard 184 were utilized to develop a flexible membrane; in this design, zinc oxide (ZnO) was added to avoid air trapping and improve adhesion. Table 7, summarizes the above-mentioned studies on semi-dry couplants.

**Figure 4:**
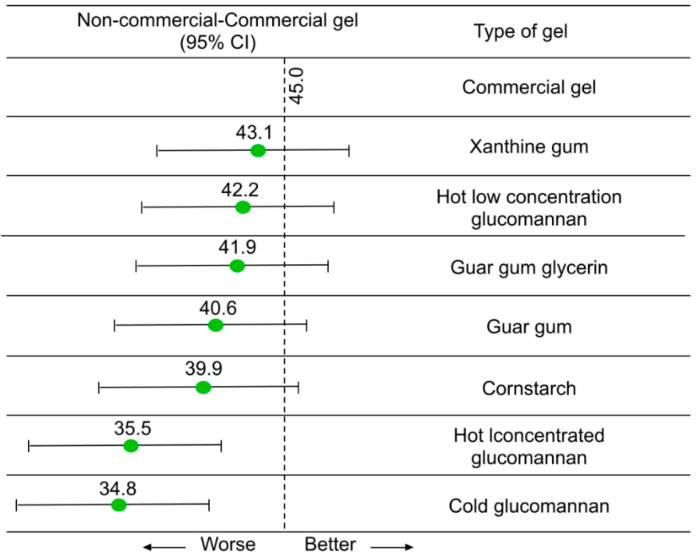
Aggregate performance of noncommercial gel alternatives compared to commercial gel. Error bars indicate adjusted 95% confidence interval (CI) from post hoc Tukey-Kramer *t-* tests; bars crossing dashed lines represent no statistically significant difference from commercial gel. The commercial gel mean score was 45 (ideal score). Reproduced from [102].

There are several non-commercial alternatives available derived from edible contents (i.e., guar gum with and without glycerin, xanthine gum, and glucomannan mixed with either hot or cold water) that can be used instead of commercial gel/water inside the enclosed assemblies. The detailed recipes are provided in [102]. This study evaluated eight different non-commercial gels made from these edible contents and evaluated their performance through ultrasound imaging. According to the authors, there is no difference in ultrasound image quality from blinded review and well suited for places where commercial gel may be unavailable, unaffordable, or both. In [102], authors have compared the non-commercial gel with commercial gel in terms of ease of use, consistency, and conformity. A total if 72 evaluations were performed by operators and for comparison purposes, a blind perfect mean score of 45 was assigned to the commercial gel. In Figure 4, the evaluation scores of the non-commercial gels are reported.

**Table 7:** Summary of semi-dry coupling materials.

Housing Material	Cross-Linking Material	Actual Couplant	Form Factor	Ref
Hydrophilic block copolymer	Biocompatible liquid	Gel	Sheath type membrane	[97]
	Polyalkylene glycol plasticizer, water, ammonium acetate, magnesium acetate	Gel	Adhesive patch	[98]
Polypropylene	-	polyurethane gel	Flexible plate	[101]
Mylar	-	Water/gel	Flexible membrane	[103]
Silicone	polyurethane, latex, and rubber	Degassed water	Rigid container	[104]
Cellulose	glycerin and water/oil	Gel	Gel pad	[105]
Sylgard 184	zinc oxide	Gel	Membrane	[106]

## 4. Coupling Mechanism within Ultrasound Transducer

**Figure 5:**
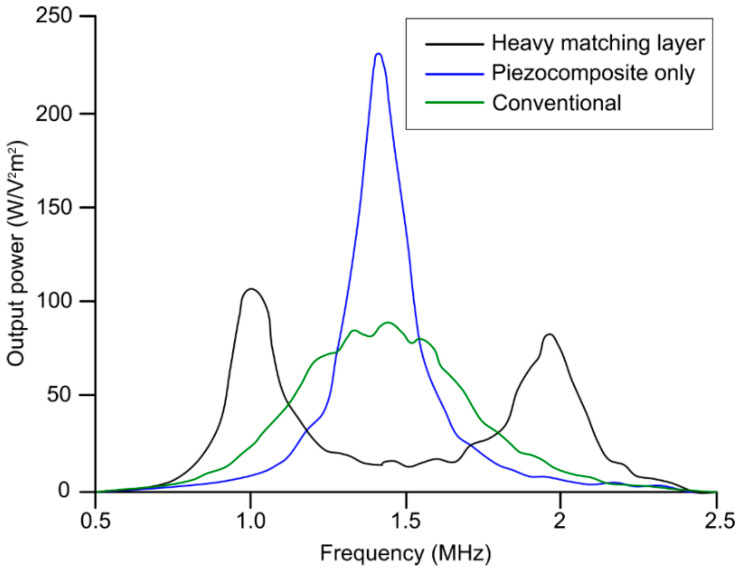
Comparison between the power generated from transducer with heavy matching layer on piezocomposite active layer, transducer without heavy matching layer on piezocomposite active layer, and transducer with conventional matching layer + piezocomposite method. Reproduced from [109].

Piezoelectric ultrasound transducers are the most widely manufactured and clinically available transducers that are integrated into commercial ultrasound imaging systems [39,54,55]. The main component of a piezoelectric ultrasound transducer is the active sensing elements constructed from piezo-material with acoustic impedance >30 MRayl [107]; this is significantly different from that of biological soft tissues. This acoustic impedance mismatch is one of main sources of reverberations in ultrasound transducers that leads to the potential misinterpretation of ultrasound b-mode images. Therefore, an additional matching layer is employed in front of active-sensing elements for an efficient propagation of the acoustic pressure wave. There are two types matching layers: (1) active and (2) passive matching layer. In active matching layers, piezoelectric material properties are altered by adding composites and nanocomposites to match directly with the propagating media. In such cases, the matched piezoelectric material itself is called active matching layer, whereas when different materials or combination of materials are used to reduce the acoustic impedance mismatch, it is called passive matching layer. A piezocomposite is a diced ceramic with polymer-filled spaces [108]. The composite has the flexibility to provide heat dissipation or structural support. The characteristic acoustic impedance is around 10 MRayl, which is much closer to water and tissue. An array of piezocomposite transducers is made by blending piezo powder, piezo-rods, or piezo fibers with various resins to simultaneously impart flexibility and higher sensitivity. The active matching technique involves the modification of piezoelectric element properties, eliminating the need for matching layers, whereas the passive matching techniques involves matching acoustic passive layers with the piezoelectric element. Typical passive matching layers are gold, aluminum, glass, and different polymers. Table 8 summarizes the acoustic properties of several matching layer materials. A heavy matching layer (increased thickness) helps to lower acoustic impedance mismatch and alters the center frequency; however, overall transducer piezoelectric characteristics eventually deteriorate and, therefore, output power decreases. Figure 5 demonstrates the impact of the matching layer (heavy and conventional thickness) on generated output power as a function of frequency compared to a transducer with no matching layer [109]. For medical imaging, a typical design approach is to consider one fourth lambda thickness for the matching layer.

## 5. Air Couplant (for Air-Coupled Transducers)

As opposed to wet, dry, or semi-dry couplants, air couplants can only be used in specially designed transducers, called air-coupled or non-contact transducers. Air-coupled transducers are made of piezoceramics with several active matching layers to reduce transducer-air impedance mismatch. In conventional ultrasound transducers, the material of the matching layers has similar acoustic impedance as the target tissue acoustic impedance. In air-coupled transducers, the materials of the matching layer are carefully chosen to closely match the acoustic impedance of air. As with dry couplants, the air couplant is mainly used during development and non-destructive testing [76] of biomedical instrumentation where a liquid or gel couplant could damage the target or transducer. Ferroelectret thin films (40~100 µm) are typically used as matching layers to produce air-coupled ultrasonic transducers because their acoustic impedance is well matched to the impedance of air [110,111]. These are heterogeneous non-polar space-charge electrets that exhibit piezoelectric response combined with mechanical flexibility and low acoustic impedance (<0.1 MRayl). Moreover, these transducers are strictly operating at the resonant frequency of sensing material (usually frequencies <500 kHz) to improve the ultrasound transmission efficiency. There have been several developments for air-coupled transducers with higher operating frequencies and improved sensitivity [112,113,114]. In Table 9, we have summarized typical specifications of air-coupled transducers that are available in the market.

## 6. Conclusions

We reviewed the characteristics of liquid/gel, semi-dry, dry acoustic, and air couplants (see the comparison between their pros and cons in Table 10). The first two parameters, impedance mismatch and attenuation, are the acoustic properties of the couplants where lower parameter values are preferable to increase acoustic wave propagation efficiency. The chemical property should allow couplants to be less degradable and simultaneously adhere more to the applied surface. The mechanical property needs to enhance the flexibility of the couplants to enable the usage on uneven or curved surfaces; however, least manufacturing complexity and eventually low production cost is preferred. We described liquid/gel couplants are the most established and widely used couplants in clinical and research bioacoustic sensing applications. We then discussed semi-dry, dry and air couplants, their acoustic properties, and how they are made. Wet couplants are best suited for biomedical ex vivo studies where immersion is feasible; for in vivo studies, semi-dry couplants are better, especially when the coupling agent is required to be suspended vertically to the imaging surface (i.e., brain, skin, or breast imaging). We explained that although dry and air couplants are feasible, their use is intended for the characterization of bioacoustic sensing instruments during development and testing.

## Figures and Tables

**Table 1:** Advantages and disadvantages of different biosensing techniques [40,41,42,43].

Biosensing Technique	Advantage	Disadvantage	Sensitivity	Selectivity
Electrochemical	- easy to integrate - label free - low cost	- mostly in-vitro - poor stability - time consuming	Low	High
Electromagnetic	- high resolution - can be non-invasive - real-time detection	- limited penetration - complex instrumentation - affected by environmental factors	High	Average
Acoustic	- label free - non-invasive - high dynamic range - deeper penetration	- limited resolution - bulky	High	Low

**Table 2:** Different form factors of acoustic coupling agents.

Type	Liquid/Gel-Based	Dry	Semi-Dry
Form factor	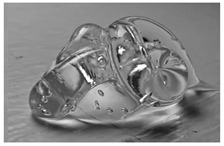	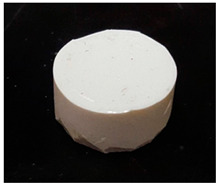	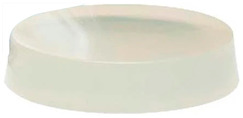

**Table 3:** Acoustic properties of water, air, and some biological tissues [57,58,59].

Material	Longitudinal Speed (m/s)	Density (g/cm^3^)	Acoustic Impedance (MRayl)	Attenuation (dB/cm·MHz)
Water	1480	1	1.5	0.0022
Air	344	0.00125	0.00001	7.5
Blood	1570	1.26	1.61	0.087
Brain	1550	1.087	1.58	0.87
Fat	1450	0.870	1.38	0.61
Liver	1590	1.06	1.69	0.9
Kidney	1570	1.05	1.65	1
Heart	1570	1.045	1.64	2
Eye lens	1525	1.04	1.72	2
Muscle	1580	1.065	1.58	0.7~1.4
Bone	3500	1.9	7.80	8.7

**Table 4:** Acoustic properties of commonly used liquid/gel-based ultrasound coupling media [63,64].

Material	Longitudinal Speed (m/s)	Density (g/cm^3^)	Acoustic Impedance (MRayl)	Attenuation (dB/cm·MHz)
Glycerin	1930	1.26	2.42	0.25
Ethylene glycol	1626	1.087	1.8	0.34
Oil	1753	0.870	1.51	0.15~0.5
Gel	1390–1620	0.98–1.03	1.45–1.60	<0.05
Water at 20 °C	1473	1	1.48	0.002

**Table 5:** Acoustic properties of conventional polymers used as dry couplants [63,64,83,84,85,86].

Type	Material	Longitudinal Speed (m/s)	Density (g/cm^3^)	Acoustic Impedance (MRayl)	Attenuation @ 5MHz (dB/cm)
Thermoplastic	PVC (soft)	2270	1.36	3.27	11.2
	PTFE	1390	2.17	3	3.9
	UHMWP	2364	0.91	2.33	8
	Polypropylene	2740	0.92	2.4	5.1
	Polycarbonate	2300	1.22	2.75	23.2
	PMMA (clear)	2750	1.20	2.32	11.3
	Nylon 6-6	2600	1.314	2.9	12.9
Thermoset	Polyester,	2290	1.21	2.86	10–20
	Epoxy	2360	1.15	2.86	15–20
Elastomer	Polyurethane	2090	0.941	2.36	27.6–100
	Polystyrene	2400	1.21	2.52	1.8
	Butadiene	1567	0.95	1.49	1
	Silicone	1041	0.99	1.04	0.71

PVC: Polyvinyl chloride; PTFE: Polytetrafluoroethylene (Teflon); UHWMP: Ultra-high-molecular weight polyethylene; PMMA: Polymethyl methacrylate; silicone: RTV12.

**Table 6:** Acoustic properties of commercially available dry ultrasound couplants [88,89,91].

Material	Type	Acoustic Speed (m/s)	Attenuation (dB/mm @5MHz)	Hardness (Shore A)	Feature
Aqualene 200	Thermoset	1589	−0.22	40	Soft, flexible
ACE 400	Thermoplastic	1541	−0.99	40	Low temperature
Aqualink	Thermoplastic	1489	0.44	5	Conforming, Clear, Supersoft
Aquasilox	Silicone based	1001	−0.80	23	High temperature
AquaCyan	Urethane	1589	−3.33	90	High abrasion, tough

**Table 8:** Acoustic properties of matching layer [107].

Material	Longitudinal Speed (m/s)	Density (g/cm^3^)	Acoustic Impedance (MRayl)
Parylene	1100	2.35	2.58
Gold	19,700	3.24	63.8
Aluminum	6320	2.70	17
Glass	5900	7.70	45
Perspex	5000	3.00	15
Anodic aluminum oxide epoxy	2350	1.06	2.5
High density polyehylene	3460	2.75	9.5
Syntactic foam	2339	0.95	2.2
Epotek 301	2486	0.70	1.75
Teflon	2800	2.30	6.4
Acrylonitrile-butadiene-styrene	2300	1.22	2.8
Polysulfone	2510	1.06	2.7
Mylar	2740	0.92	2.4

**Table 9:** Specification of the air-coupled transducer [115,116].

Transducer Diameter (mm)	Center Frequency (kHz)	Sensing Range (m)
205	19.5	0.8–40
106	30	0.8–25
77.5	41	0.35–15
77.5	50	0.30–10
76.2	75	0.25–7
25	125	0.20–3
16	200	0.12–2
13	228	0.10–1.5
12	300	0.05–0.5

**Table 10:** Qualitative comparison between different acoustic couplants.

Parameters	Liquid/Gel	Dry	Semi-Dry	Air-Coupled
Impedance mismatch	+	++++	++	++++
Attenuation	+	++++	++	++++
Biodegradability	++++	+	++	+
Flexibility	++++	+	+++	++++
Adhesion	++	+	+++	-
Cost	++	+	+++	+++
Complexity (Develop)	+	+++	++++	++++
Complexity (Usage)	+	++	+++	+

++++: high, +++/++: medium, +: low, - Not applicable.

## Data Availability

Not Applicable.
